# Beyond Dementia: A Case Series of Primary Delusional Parasitosis in Cognitively Intact Octogenarians From North India

**DOI:** 10.7759/cureus.108013

**Published:** 2026-04-30

**Authors:** Dhiraj Raja, Shikha Singh, Alankrita Singh

**Affiliations:** 1 Department of Psychiatry, Heritage Institute of Medical Sciences, Varanasi, IND

**Keywords:** delusional infestation, delusional parasitosis, geriatric psychiatry, late-onset psychosis, somatic delusion

## Abstract

Delusional parasitosis is an uncommon psychiatric condition in which a person strongly believes that insects or parasites are present on or inside the body, even when there is no medical evidence to support it. Because the sensations feel real, most patients first approach dermatologists and only later come to psychiatric services. In older patients, these symptoms are often taken as part of a medical or neurological illness, such as dementia, so the possibility of a primary psychiatric condition may be overlooked. In this case series, we describe three patients aged 80 years or older who presented with similar complaints of crawling sensations, continuous itching, and a fixed belief that something was present on their bodies. All of them had already consulted several doctors before reaching psychiatry. On examination, they were alert, oriented, and able to communicate well, with only mild age-related cognitive changes that were not sufficient to diagnose dementia. Routine investigations, including blood tests, brain imaging, and dermatological evaluation, were normal in all cases. Treatment with second-generation antipsychotics led to some improvement in symptoms such as itching, sleep, and distress, but the main belief persisted. These cases suggest that, even in very old age, such symptoms are not always due to an underlying medical or brain disorder and may represent a primary psychiatric illness.

## Introduction

Delusional parasitosis is a condition in which a person strongly believes that insects or parasites are living on or inside their body, even when there is no medical evidence to support it [1]. It is considered a somatic type of delusional disorder, where the belief stays fixed and does not change even after repeated reassurance [2]. In clinical practice, these patients rarely present to psychiatry first. Most of them initially consult dermatologists because the symptoms feel physical, such as itching, crawling sensations, or a feeling of movement on the skin [3]. They often move from one doctor to another before finally reaching psychiatric care [4].

This condition is quite rare, with an estimated prevalence of about 20-40 cases per million population [5]. It is more commonly reported in older age groups and has been described in clinical case series highlighting its persistent nature [6]. In elderly patients, these symptoms are often attributed to a physical or neurological problem, such as dementia or another neurological illness [7]. Because of this, there is a strong tendency to assume that the problem is "organic" rather than a primary psychiatric condition.

In day-to-day practice, these patients present with a very fixed belief that something is infesting their body. Along with this, they experience sensations, such as crawling or itching. Many of them also start collecting small things, such as skin flakes or dust, believing this is proof of what is happening to them [8].

Because of this strong conviction and poor insight, patients often continue dermatological consultations and delay psychiatric treatment [3,4].

Indian literature shows similar patterns but is largely limited to small case reports and case series [9,10]. There is very limited data on patients aged 80 years or older without identifiable organic causes. This case series attempts to address this gap.

## Case presentation

Three elderly patients, all over 80 years of age, presented to our psychiatry department with a very similar complaint. All of them described a strange and persistent feeling that something was crawling or moving on or under their skin. Before coming to psychiatry, each of them had already consulted different departments, especially dermatology. Even after multiple evaluations, no clear medical cause could be found in any of the cases.

Consent

Written informed consent was obtained from the patients and/or their legal guardians for publication of this case series. All efforts were made to ensure patient anonymity.

Case 1

A 92-year-old woman slowly walked into the clinic, holding a small steel glass. She looked calm, but there was clear discomfort on her face. As soon as she sat down, she said, "Doctor, something is crawling under my skin." She placed the glass on the table and showed what was inside. There were small flakes of peeled skin. For her, these were not just skin particles; she believed this was the actual problem.

For nearly two years, she had been feeling as if something was constantly crawling under her skin. Because of this, she kept scratching herself repeatedly. Whatever small bits of skin came off her, she carefully collected, believing that they were responsible for her symptoms. She had already seen many doctors and had taken medications, including sertraline, for about three months, but nothing helped.

During assessment, she was alert and fully oriented. Cognitive functions were assessed clinically and did not reveal significant impairment suggestive of dementia. All her investigations were normal. Blood tests were within normal limits, brain imaging showed only age-related changes, and skin examination did not reveal any infection or infestation. Despite reassurance and the lack of supporting medical evidence, her belief remained firm. What stood out most was her certainty. She did not question her experience at all.

Case 2

An 86-year-old man came with a slightly different but equally strong complaint. He placed his hand on his abdomen and said, "It starts here in the stomach and then spreads all over my body." He described a sensation that began in his abdomen and gradually spread to his limbs and back. It was not just itching; he clearly felt something moving inside him.

He had a history of irritable bowel syndrome for eight years and hypertension for 15 years. He had been prescribed escitalopram earlier but stopped it on his own, saying it did not help.

During the interview, he kept rubbing his abdomen and scratching his arms and legs almost continuously. Even after consulting several doctors and being told that everything was normal, he continued to believe that something was wrong inside his body. He had also become more irritable, and his sleep was disturbed.

On examination, he was alert, oriented, and able to communicate clearly. Cognitive functions were assessed clinically and did not reveal significant impairment suggestive of dementia. All investigations again came back normal. Blood reports were within normal limits, brain imaging showed only age-related changes, and skin examination was normal. There was no medical explanation, but for him, what he was feeling was completely real.

Case 3

An 83-year-old woman was brought by her grandson, who seemed worried. He said, "Doctor, she keeps saying something is crawling on her. She doesn't sleep and keeps scratching all the time." When asked, she said, "They are moving on my back all the time."

For about three years, she had been experiencing continuous crawling and tingling sensations over her back, hands, and legs. She had first visited a dermatologist, where no problem was found, and was later referred to a psychiatrist. She had taken antihistamines for some time, but did not improve.

During the examination, she appeared restless and kept trying to scratch her back. There were visible scratch marks on her back and legs. Her belief was very strong. She was convinced that insects were moving on her body. Insight was poor, and her judgment was affected.

Her thinking and memory were assessed clinically and did not show significant impairment suggestive of dementia. All her reports were normal. Blood tests were within normal limits, the brain scan showed only age-related changes, and the skin examination did not reveal any abnormality. Even after explaining things to her multiple times, she continued to feel the same and remained strongly convinced about it.

When we look at all three patients together, a similar pattern becomes clear. All of them were convinced that something was affecting their body. They described sensations such as crawling, itching, or something moving under the skin.

Before reaching psychiatry, all of them had visited dermatology multiple times, but no physical cause was found. Their cognitive changes were mild and not enough to explain these symptoms as dementia. After treatment, there was some improvement in sleep and overall distress, but the main belief did not completely go away.

What ties these cases together

These patients were not confused or disoriented. They knew who they were, where they were, and could communicate clearly. Still, they remained fully convinced about something that could not be explained medically. That is what makes these cases important, both clinically and in day-to-day practice.

All patients met the International Classification of Diseases, 11th Revision (ICD-11) criteria for persistent delusional disorder (somatic subtype). Detailed evaluation ruled out medical, neurological, and dermatological causes. Treatment with second-generation antipsychotics, mainly risperidone, led to partial improvement, although the core belief continued, which is in line with what has been reported in earlier studies [4,5]. A summary of the clinical characteristics, treatment, and outcomes of all cases is provided in Table 1.

**Table 1:** A summary of the clinical characteristics, treatment, and outcomes of all three cases. All patients met the International Classification of Diseases, 11th Revision (ICD‑11) criteria for persistent delusional disorder (somatic subtype). Comprehensive evaluations, including blood investigations, brain imaging, and dermatological examination, were normal in all three cases. Summary of clinical characteristics, treatment history, and outcomes of three elderly patients with primary delusional parasitosis. All patients showed partial improvement in distress and sleep with antipsychotic treatment, while the core delusional belief persisted despite adequate clinical evaluation and management.

Case	Age (years)	Gender	Duration of symptoms	Key symptoms and clinical features	Past treatment history (before psychiatry)	Treatment at our psychiatry department	Outcome
1	92	Female	~2 years	Persistent sensation of something crawling under the skin; repeated scratching; collection of peeled skin flakes, believed to be the cause; firm, unshakable belief despite reassurance	Multiple dermatology consultations; treated with sertraline for ~3 months - no benefit	Started on second-generation antipsychotic (risperidone)	Partial improvement in sleep and overall distress; core belief persisted
2	86	Male	Not clearly defined (chronic)	Sensation begins in the abdomen and spreads to the whole body; a feeling of something moving inside; continuous scratching of the abdomen, arms, and legs; irritability and sleep disturbance	Multiple medical consultations; history of irritable bowel syndrome (8 years) and hypertension (15 years); previously prescribed escitalopram - stopped on his own (no benefit)	Started on second-generation antipsychotic (risperidone)	Partial improvement in symptoms and sleep; core belief persisted
3	83	Female	~3 years	Crawling and tingling sensations over the head, hands, and legs; continuous scratching, especially over the back; visible scratch marks; strong conviction of insects crawling; poor insight and affected judgment	Dermatology consultation - no abnormality found; treated with antihistamines - no benefit	Started on second-generation antipsychotic (risperidone)	Some improvement in sleep and distress; belief persisted

## Discussion

In this case series, we observed that primary delusional parasitosis can occur in cognitively intact elderly individuals and may present with significant distress despite the absence of underlying organic pathology. All patients showed improvement with low-dose atypical antipsychotics and supportive measures.

Global perspective

Delusional parasitosis is a rare condition, seen in only about 20-40 people per million, but it causes significant distress to the patient [5]. Clinical case series have described its persistent nature and the challenges associated with management [6]. In older patients, there is a common tendency to link these symptoms to medical or neurological conditions, such as dementia [7], which may delay consideration of a primary psychiatric diagnosis. In real-world clinical settings, these patients rarely present to psychiatry first. They go to dermatologists because what they feel seems very physical, such as itching, crawling, or something moving on the skin [3]. Many of them keep going from one doctor to another, trying to find a reason. Only later, often after several consultations, do they reach psychiatric care [4]. This delay is significant because it increases suffering and strengthens the belief over time.

Indian perspective

In India, most available data come from case reports and small case series [9,10]. Even though studies are limited, the pattern observed is quite similar to that reported globally. Patients usually first visit dermatologists; there is a delay before they are referred to psychiatry, and treatment leads to only partial improvement rather than complete recovery. This shows the real challenge in practice. Patients may feel better in terms of sleep and distress, but the belief itself often continues in some form [11,12]. At the same time, there is very little data specifically focusing on patients aged 80 or older, especially those without a clear medical or neurological cause. This creates an important gap in understanding this condition in very elderly patients.

What is unique about our case series

Our cases highlight some simple but important points. All three patients were above 80 years of age, yet none of them had dementia or any clear medical or neurological illness that could explain their symptoms. Their presentation was quite typical, with a strong fixed belief and clear sensations, such as crawling or itching, which match known clinical features [8].

They also followed a pattern similar to that seen in real practice, with multiple consultations, especially in dermatology, before reaching psychiatry [3,4]. Treatment led to some improvement in sleep, distress, and discomfort, but the main belief did not fully go away. This is similar to what has been seen in other treatment studies [11,12]. Even though the number of cases is small, their similarity makes the observation meaningful. What stands out is something very simple but important: even at a very advanced age, this condition can exist on its own, without any underlying organic cause.

Clinical insight

These cases highlight an easy-to-miss aspect of day-to-day practice. Advanced age does not always mean that symptoms are due to dementia or other organic causes. Careful assessment is needed before labeling a condition as organic. There is also a need for better coordination between dermatology and psychiatry, because these patients often move between specialties without getting a clear answer. In simple terms, not every elderly patient with such symptoms has a brain disease. Sometimes, it is a psychiatric illness presenting later in life.

Limitations

This case series has a few limitations. The number of patients is small, and we could not follow them for a long period. Because of this, it is difficult to understand how the condition changes over time. More studies with larger patient numbers are needed to better understand this condition in very elderly individuals.

## Conclusions

Delusional parasitosis is commonly associated with middle age and, when seen in the elderly, is often attributed to underlying medical or neurological conditions. However, this case series demonstrates that it can occur as a primary psychiatric disorder even in advanced age without identifiable organic causes. These findings challenge the routine assumption that late-onset symptoms are always secondary to brain pathology and emphasize the importance of thorough clinical evaluation before attributing such presentations to neurocognitive disorders.
